# Selection and Validation of Endogenous Reference microRNAs for Post-Mortem Interval Estimation in Vitreous Humor: A Preliminary Study

**DOI:** 10.3390/ijms27052102

**Published:** 2026-02-24

**Authors:** Julia Lazzari, Andrea Scatena, Marco Di Paolo, Anna Rocchi

**Affiliations:** Institute of Legal Medicine, Department of Surgical Pathology, Medical, Molecular and Critical Area, University of Pisa, Via Roma 55, 56126 Pisa, Italy; dott.andrea.scatena@gmail.com (A.S.); marco.dipaolo@unipi.it (M.D.P.); anna.rocchi@unipi.it (A.R.)

**Keywords:** forensic science, post-mortem interval (PMI), vitreous humor, microRNA, reference gene, qPCR normalization

## Abstract

Estimating the post-mortem interval (PMI) using microRNAs (miRNAs) in vitreous humor (VH) is a promising technique in forensic pathology. However, the reliability of quantitative Real-Time PCR (qPCR) data in this matrix is currently constrained by a critical methodological challenge: the lack of a rigorously validated endogenous reference gene (normalizer) capable of correcting for non-biological variations without being influenced by decomposition. This study aimed to identify a robust reference gene for VH analysis by performing a comparative validation of two candidates proposed in the literature: miR-222-3p and miR-96-5p. VH samples were collected from 47 forensic autopsy cases with estimated PMIs ranging from 3 to 24 h. The validation process assessed three key parameters: amplification detectability, expression stability (Coefficient of Variation, CV), and statistical independence from both the PMI and the pre-analytical freezing interval using regression models. MiR-222-3p was rejected as a normalizer due to poor detectability, failing to reach the detection threshold (Cq < 35) in 61.7% of cases (29/47). Conversely, hsa-miR-96-5p was validated as a stable reference gene. It demonstrated high detectability and expression stability (CV = 9.07%) among valid samples. Crucially, linear regression analysis showed no significant correlation between hsa-miR-96-5p levels and either the PMI (*p* = 0.69) and the pre-freezing time (*p* = 0.70). This study demonstrates that miR-222-3p is unsuitable for forensic casework in VH due to instability. We identified and validated hsa-miR-96-5p as a robust endogenous reference gene. Its adoption is recommended to standardize future molecular thanatochronology studies and improve the accuracy of PMI estimation models.

## 1. Introduction

The accurate estimation of the Post-Mortem Interval (PMI) remains one of the most complex and critical challenges in forensic pathology. Determining the time since death is crucial for both criminal investigations, to verify alibis or reconstruct events, and civil matters, such as succession or insurance claims.

Traditionally, PMI estimation relies on the assessment of consecutive abiotic phenomena, such as algor mortis, rigor mortis, and livor mortis. Among biochemical methods, the analysis of potassium concentration (K^+^) in the vitreous humor (VH) is considered the gold standard due to the anatomical isolation and relative sterility of the vitreous chamber [1,2]. However, these traditional methods are heavily influenced by extrinsic variables—primarily ambient temperature—and often yield confidence intervals that are too wide to be decisive in judicial contexts.

Forensic research regarding PMI estimation has focused on the ‘molecular clock’ hypothesis [3], identifying miRNAs as ideal candidates due to their high post-mortem stability, particularly within the VH [4,5,6]. However, the practical application of this technique via RT-qPCR encounters a critical methodological obstacle: data normalization. Indeed, it is crucial to identify an endogenous reference gene that is not only validated but whose expression remains stable independent of decomposition processes and the post-mortem interval [7].

Currently, there is no consensus in the literature regarding a validated reference gene for miRNA analysis in post-mortem vitreous humor. Previous pilot studies have proposed candidates such as miR-222-3p or miR-96-5p, but results have been contradictory or inconclusive [8,9,10]. Specifically, we selected miR-222-3p because it was identified as a stable reference by Odriozola et al. [8], and miR-96-5p due to its high expression in VH reported by Corradini et al. [9]. Using an unvalidated reference gene poses a significant risk of misinterpreting data, as the degradation of the normalizer itself could mask or exaggerate the degradation of the target markers.

Therefore, the primary aim of this study was to perform a rigorous comparative validation of two candidate endogenous reference genes, miR-222-3p and miR-96-5p, in vitreous humor samples collected from forensic autopsies. We evaluated their detectability, expression stability, and statistical independence from both the PMI and pre-analytical freezing times. This work seeks to establish a robust methodological standard for future molecular thanatochronology studies involving vitreous humor.

## 2. Results

### 2.1. Sample Characteristics and Quality Assessment

A total of 47 vitreous humor samples were initially collected and subjected to molecular analysis. Following the quality control phase based on the amplification of candidate reference genes, 27 samples (57.4%) were deemed valid for final quantification. The valid cohort (*N* = 27) consisted of 14 males and 13 females, with a mean age of 51.4 years (range: 22–84 years). The causes of death were heterogeneous, comprising gunshot wounds, drug overdoses, and various forms of mechanical asphyxia, all united by the requirement for a judicial autopsy. The excluded samples failed to show reliable amplification signals (Cq ≥ 35) for the designated reference genes. A retrospective analysis of these excluded cases revealed no specific clustering regarding PMI or age, and ocular pathologies had been ruled out by inclusion criteria. Thus, the exclusion was attributed to the intrinsic low RNA yield of the vitreous matrix and pre-analytical variability. In the final valid cohort (*N* = 27), the estimated Post-Mortem Interval (PMI) ranged from 3.0 to 24.0 h, with a mean of 11.88 ± 7.03 h. The pre-analytical interval (time from collection to freezing) varied between 30 and 600 min, with a mean of 154 ± 127.4 min (Table 1).

### 2.2. Evaluation of hsa-miR-222-3p: Detectability Failure

**Figure 1:**
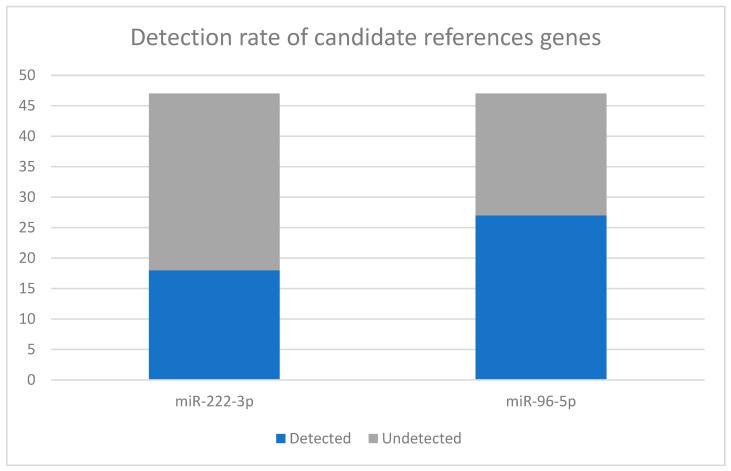
Detection rates of candidate reference genes in vitreous humor (*N* = 47). The stacked bar chart illustrates the superior detectability of miR-96-5p (57.4% detection rate) compared to miR-222-3p (38.3% detection rate). Blue bars represent samples with reliable amplification (Cq < 35), while grey bars represent undetected or failed samples.

Based on the previous literature proposing miR-222-3p as a reference gene for vitreous humor, this candidate was the first to be evaluated. However, analysis of the raw amplification data across the entire initial cohort (*N* = 47) revealed significant instability. As shown in Figure 1, miR-222-3p was consistently detected (Cq < 35) in only 18 out of 47 samples (38.3%). In the remaining 61.7% of cases, the target was either undetermined or fell below the limit of detection. Due to this high rate of amplification failure, miR-222-3p was rejected as a universal normalizer for this specific matrix.

### 2.3. Validation of hsa-miR-96-5p: Stability and Independence

Consequently, the performance of hsa-miR-96-5p was evaluated. This candidate showed a significantly higher detection rate, being reliably amplified in 27 samples, which constituted the final study cohort

#### 2.3.1. Absolute Expression Stability

To assess the expression stability of miR-96-5p, we analyzed the dispersion of its raw Cq values across the 27 valid samples. The candidate exhibited a mean Cq of 30.21 with a standard deviation of 2.74. The calculated Coefficient of Variation (CV) was 9.07%. This CV value falls within the generally accepted range (<10–15%) for reference genes in heterogeneous biological samples, indicating robust absolute stability despite inter-individual variability.

#### 2.3.2. Independence from PMI and Pre-Analytical Variables

**Figure 2:**
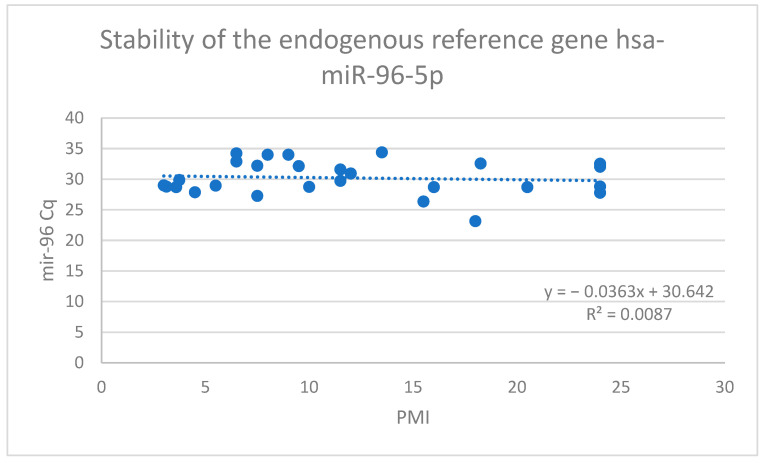
The scatter plot correlates the raw Cq values (Y-axis) of the valid samples (*N* = 27) with their estimated Post-Mortem Interval (X-axis). The linear regression analysis confirms the absence of significant correlation (*p* = 0.690), demonstrating that miR-96-5p expression is independent of the decomposition time within the analyzed window (3–24 h).

The critical requirement for a forensic normalizer is statistical independence from the post-mortem interval. We performed a multiple linear regression analysis to test the correlation between the Cq values of miR-96-5p (dependent variable) and two temporal variables: PMI and the collection-to-freezing time. The analysis revealed no significant correlation with either variable. As illustrated in Figure 2, the regression yielded a *p*-value of 0.690 for the PMI and a *p*-value of 0.696 for the freezing time. These values (*p* > 0.05) indicate a failure to reject the null hypothesis, confirming that the expression levels of miR-96-5p are statistically independent of the decomposition time and sample handling delays within the investigated timeframe.

### 2.4. Selection of the Reference Standard

Based on the comparative analysis, miR-222-3p was discarded due to poor detectability. Conversely, miR-96-5p demonstrated high detectability, low variation (CV = 9.07%), and complete statistical independence from PMI (*p* = 0.69). Therefore, miR-96-5p was selected and validated as the endogenous reference gene for the normalization of target miRNAs in this study (Table 2).

## 3. Discussion

The application of microRNA (miRNA) profiling to forensic pathology represents a promising frontier for the estimation of the Post-Mortem Interval (PMI). However, the reliability of quantitative Real-Time PCR (RT-qPCR) in this context is fundamentally contingent upon to the accurate normalization of data. While vitreous humor (VH) is often described as a “biological sanctuary” that delays putrefaction compared to other matrices, the lack of a rigorously validated endogenous reference gene has hindered the standardization of molecular thanatochronology. This study addressed this critical methodological gap by performing a comparative validation of two candidate normalizers, hsa-miR-222-3p and hsa-miR-96-5p, on a cohort of real forensic casework samples.

### 3.1. The Unsuitability of miR-222-3p

The primary finding of this study is the unsuitability of miR-222-3p as a universal normalizer for post-mortem vitreous humor. This contrasts with earlier pilot studies, such as the one by Odriozola et al. [8], which identified miR-222 as a stable reference gene. However, our data revealed a critical limitation regarding its detectability in real-world forensic scenarios. Analysis of the initial cohort (*N* = 47) showed that miR-222-3p was reliably detected (Cq < 35) in only 38.3% of cases. In the remaining 61.7%, the target was either undetermined or fell below the limit of detection. This high rate of amplification failure suggests that miR-222-3p expression levels may be too low to withstand the degradation processes inherent to forensic samples, or that its stability is compromised over longer PMIs compared to controlled experimental conditions. Consequently, relying on miR-222-3p poses a significant risk of data loss and statistical bias, justifying its exclusion from our validation protocol.

### 3.2. Validation of miR-96-5p as a Robust Standard

In contrast to the failure of miR-222-3p, hsa-miR-96-5p emerged as a highly robust endogenous reference gene. It demonstrated superior detectability, allowing for the retrieval of a significantly larger proportion of the cohort for analysis.

The validation of miR-96-5p was supported by two key lines of evidence:

1. Robust Stability: The candidate exhibited a Coefficient of Variation (CV) of 9.07% across the valid samples. According to MIQE guidelines [7], a CV below 15% is generally considered acceptable for reference genes in heterogeneous biological samples. Achieving such stability in post-mortem tissues, which are subject to uncontrolled environmental variables, confirms the resilience of this marker.
2. Independence from PMI: The fundamental requirement for a thanatochronological reference gene is that its expression must not correlate with the time since death. Our regression analysis confirmed that miR-96-5p levels are statistically independent of the PMI (*p* = 0.690). This “flat” expression profile ensures that any variations observed in target miRNAs (such as degradation or accumulation) are biological realities and not artifacts of normalization.

### 3.3. Robustness Against Pre-Analytical Variables

Another significant advantage of miR-96-5p identified in this study is its independence from pre-analytical variables. Forensic fieldwork often involves unavoidable delays between sample collection and storage. Our analysis showed no significant correlation between miR-96-5p expression, and the time elapsed between collection and freezing (*p* = 0.696). This robustness suggests that miR-96-5p can serve as a reliable normalizer even in cases where immediate cryopreservation is not feasible, a common scenario in routine medicolegal practice.

### 3.4. Study Limitations

While these results are encouraging, some limitations must be acknowledged, characterizing this work as a preliminary study. The sample size (*N* = 27 valid samples), although sufficient to demonstrate statistical significance in this pilot study, warrants validation on a larger, multi-centric cohort. A critical observation in this study was the high rate of sample exclusion during the quality control phase. Out of the initial 47 cases, 20 samples were deemed unsuitable for final quantification because they failed to show reliable amplification signals. Future research must specifically focus on elucidating the underlying motivations for these exclusions. It remains to be determined whether such failures are linked to advanced biological degradation, variations in the chemical concentration of the vitreous matrix, or external environmental factors encountered during the pre-analytical phase. Although exogenous synthetic spike-in control was not employed to monitor extraction efficiency, the consistent amplification of the endogenous reference gene (miR-96-5p) across the valid samples served as an internal confirmation of successful RNA recovery and PCR competence. Additionally, the inherent uncertainty in estimating the PMI from circumstantial data introduces a variable that could be mitigated in future studies by including cases with more precisely documented times of death.

Finally, regarding practical application, a direct cost-effective comparison with standard biochemical methods (e.g., potassium analysis) was not performed in this study. As this is a preliminary investigation, the current costs reflect an experimental setting and cannot be reliably extrapolated to a standardized forensic routine. Future studies focusing on protocol optimization and standardization will be necessary to accurately assess the economic feasibility and competitive advantage of this technique in everyday casework.

## 4. Material and Methods

### 4.1. Study Design and Sample Collection

This prospective observational study analyzed vitreous humor (VH) samples collected during medicolegal death scene investigation. The initial cohort consisted of 47 cases. Samples were collected via trans-scleral puncture (oculocentesis) using a sterile syringe to aspirate approximately 1 mL of vitreous fluid from a single eye, ensuring total asepsis to prevent contamination; subsequently, an aliquot of 400 µL was processed for RNA extraction.

Following collection, samples were transferred to sterile tubes and stored at −20 °C until molecular analysis to preserve nucleic acid integrity. For each case, the “pre-freezing interval” (time elapsed between sample collection and freezing) was recorded to account for ex vivo degradation variables. Exclusion criteria included cases with ocular trauma or known ophthalmic pathologies that could alter the vitreous composition.

### 4.2. Post-Mortem Interval (PMI) Estimation

The PMI was estimated based on available circumstantial data, including police reports, witness statements, and medical records. The reliable time window was defined between the “Last Seen Alive” (LSA) and the “Discovery of Body” (DoB) or confirmation of death. For statistical correlation purposes, a point value for the PMI was calculated as the mean of this uncertainty interval.

### 4.3. RNA Extraction and Quality Control

Total RNA, enriched for small RNAs, was extracted from 400 µL of vitreous humor using the miRNeasy Mini Kit (Qiagen, Hilden, Germany), following the manufacturer’s protocol. The concentration and purity of the extracted RNA were assessed using a NanoDrop™ 2000 Spectrophotometer (Thermo Fisher Scientific, Waltham, MA, USA), measuring absorbance at 260 nm and evaluating A260/A280 and A260/A230 ratios. RNA extracts were stored at −80 °C until further processing.

### 4.4. Reverse Transcription and Quantitative Real-Time PCR (RT-qPCR)

Reverse transcription (RT) of RNA into cDNA was performed using the TaqMan^®^ Advanced miRNA cDNA Synthesis Kit (Applied Biosystems, Foster City, CA, USA). Negative controls (no template) were included in each RT session.

Quantitative analysis was conducted to compare two candidate endogenous reference genes: hsa-miR-222-3p and hsa-miR-96-5p. Real-Time PCR was performed using TaqMan Fast Advanced Master Mix and specific TaqMan Advanced miRNA Assays (hsa-miR-96-5p: Assay ID 478215; hsa-miR-222-3p: Assay ID 477982; Applied Biosystems) on a QuantStudio™ 5 Real-Time PCR System. Reaction conditions were strictly maintained according to the manufacturer’s standardized protocol to ensure reproducibility. No modifications to the reaction chemistry were applied to artificially enhance the limit of detection (LOD), prioritizing specific target amplification over the risk of detecting background noise. All reactions were performed in technical duplicates. Raw quantification cycle (Cq) values were determined using the QuantStudio™ Design & Analysis Software version 1.6.1 (Thermo Fisher Scientific) with automatic baseline and threshold settings.

### 4.5. Statistical Analysis: Validation Strategy

The core of this study was the validation of a reliable normalizer. Data analysis was performed in three sequential steps to assess the suitability of miR-222-3p and miR-96-5p:

1. Detectability Test: An initial frequency analysis was conducted on the total cohort (*N* = 47) to determine the detection rate. Samples with Cq ≥ 35 or “Undetermined” were treated as missing data (amplification failure) and excluded. Consequently, candidates with high failure rates were excluded from further validation complete statistical independence
2. Expression Stability Assessment: For the candidate meeting detectability criteria, expression stability was evaluated by calculating the Coefficient of Variation (CV) of raw Cq values across all valid samples. A lower CV indicates higher stability across the population.
3. Independence Test (Regression Analysis): To ensure the reference gene was not affected by decomposition, a multiple linear regression model was constructed. The Cq value of the candidate (dependent variable) was tested against two independent variables: PMI (minutes) and Pre-Freezing Time (minutes). A valid normalizer must show no statistically significant correlation (*p* > 0.05) with either temporal variable, confirming that its expression is independent of the post-mortem interval.

## 5. Conclusions

This study established a rigorous methodological framework for the molecular estimation of the post-mortem interval in vitreous humor by addressing the critical lack of validated normalization strategies. Our findings led to the rejection of miR-222-3p as a reliable normalizer for forensic casework because of its excessively high rate of detection failure. In contrast, miR-96-5p was successfully validated as a robust reference standard, demonstrating high detectability, acceptable expression stability, and complete statistical independence from both the PMI and pre-analytical storage conditions.

It is important to note that the rigorous selection criteria applied in this preliminary study, while necessary to ensure robust validation data, resulted in a high exclusion rate. Having now established substantial baseline data, future multicentric studies will be able to expand the cohort and specifically investigate whether these exclusions correlate with specific biological clusters (e.g., advanced age or prolonged PMI) that were not statistically evident in this pilot phase. Moreover, it is essential to perform a dedicated study to understanding the biological and technical reasons why certain samples fail to amplify, ensuring a more comprehensive understanding of miRNA stability in post-mortem tissues. Additionally, while this study confirmed the stability of miR-96-5p within the first 24 h, future investigations should aim to extend the observational window (e.g., up to 72 h or more) to verify if the marker’s stability persists during advanced decomposition stages. By identifying stable endogenous control, this work provides a foundation for the implementation of miR-96-5p in future RT-qPCR profiling, which is crucial for advancing practical forensic applications and the accuracy of PMI estimations.

## Figures and Tables

**Table 1:** Descriptive statistics of the study cohort (*N* = 27). The table summarizes the estimated Post-Mortem Interval (PMI) and the pre-analytical interval (time elapsed between sample collection and freezing). n.a.: not applicable.

Variable	Mean	SD	Median	Range (Min–Max)
PMI (hours)	11.88	7.04	11.5	3.0–24.0
Pre-freezing Time (minutes)	154.0	127.4	n.a.	30–600

**Table 2:** Comparative validation of candidate endogenous reference genes in vitreous humor.

Candidate miRNA	Detectability Rate (N = 47)	Stability (CV) (N = 27)	Correlation with PMI (*p*-Value)	Validation Outcome
hsa-miR-222-3p	38.3% (18/47)	Not assessed	Not assessed	Rejected
hsa-miR-96-5p	57.4% (27/47)	9.07%	0.690	Validated

## Data Availability

The data presented in this study are available on request from the corresponding author. The data are not publicly available due to investigative secrecy.
